# The SWI/SNF complex in tumor metabolism: Mechanisms and therapeutic implications

**DOI:** 10.1016/j.jbc.2026.111342

**Published:** 2026-03-04

**Authors:** Xuan-Hao Pan, Jian Wang, Jing Su, Rui Zhao, Yu-Fei Gao

**Affiliations:** 1 Department of Neurosurgery, China−Japan Union Hospital of Jilin University, Changchun, Jilin, China; 2 Jilin Province Neuro-Oncology Engineering Laboratory, Changchun, Jilin, China; 3 Jilin Provincial Key Laboratory of Neuro-Oncology, Changchun, Jilin, China; 4 Key Laboratory of Pathobiology, Ministry of Education, Department of Pathophysiology, College of Basic Medical Sciences, Jilin University, Changchun, Jilin, China; 5 Department of Urology, China−Japan Union Hospital of Jilin University, Changchun, Jilin, China

**Keywords:** cancer metabolic reprogramming, epigenetic regulation, glutamine metabolism, glycolysis, oxidative phosphorylation, SWI/SNF complex

## Abstract

Cancer metabolic reprogramming is a driver of tumorigenesis and progression. While extensive research has highlighted the roles of metabolic enzymes and signaling pathways in this process, the mechanisms by which chromatin regulation coordinates the metabolic network at the transcriptional level remain unclear. The SWI/SNF chromatin remodeling complex, a key epigenetic regulator, has recently been shown to modulate multiple tumor metabolic pathways. Metabolic reprogramming induced by mutations in its subunits has garnered increasing attention, but comprehensive reviews on how SWI/SNF-mediated chromatin remodeling governs this process are limited. This paper examines how the SWI/SNF complex regulates metabolic gene transcription by positioning promoters and enhancer regions, guided by transcription factors, and remodeling nucleosome structures. It further discusses its role in regulating glycolysis, the tricarboxylic acid cycle, oxidative phosphorylation, lipid metabolism, and the coupling of carbon-nitrogen metabolism between amino acids and glucose-lipid metabolism. Focusing on subunit mutations such as ARID1A, SMARCA4, and PBRM1, this paper explores their impact on metabolic adaptation, offering insights for identifying therapeutic targets. Based on these findings, a combination intervention strategy targeting the protein levels of glutaminase 1, oxidative phosphorylation (complex I), glutamine transport, and glycolysis is proposed. By integrating SWI/SNF complex status and metabolic phenotypes, a therapeutic framework is developed that balances metabolic compensation blockade and enhanced cell death sensitivity, providing a more precise treatment strategy for metabolism-dependent tumors.

Metabolism is the fundamental physiological process that sustains the growth, development, and survival of organisms. It provides cells with energy sources like ATP and biosynthetic substrates, supporting cell growth, differentiation, stress responses, and homeostasis through the coordinated interactions between glycolysis, TCA cycle, oxidative phosphorylation (OXPHOS), and lipid and amino acid metabolism (1, 2, 3). Recent systematic reviews and multi-omics studies have highlighted the critical regulatory roles of these metabolic networks in maintaining health and disease development (4, 5). For instance, in various cancers, the PI3K–Akt–mTORC1, c-Myc, and hypoxia-induced HIF-1α signaling pathways cooperate to promote GLUT1 expression and membrane localization, thereby enhancing glucose uptake capacity. Simultaneously, the upregulation of the rate-limiting glycolytic enzymes HK2 and phosphofructokinase 2/fructose-2,6-bisphosphatase 3, the latter of which activates Phosphofructokinase 1 by regulating the levels of fructose-2,6-bisphosphate, results in the preferential expression of the PKM2 isoenzyme. These changes collectively drive glycolysis into a metabolic state that supports biosynthesis. Consequently, carbon flux is redirected into the pentose phosphate pathway (PPP) to generate NADPH, which meets ATP energy requirements for proliferation and enhancing resistance to oxidative stress and apoptotic signals (6, 7). Furthermore, within the tumor immune microenvironment, tumor-associated macrophages (TAMs) often exhibit increased fatty acid oxidation (FAO) and mitochondrial OXPHOS activity to maintain their immunosuppressive phenotype. Under hypoxic conditions and lactate accumulation, TAMs activate glycolysis through HIF-1α-mediated signaling pathways, thus shifting their metabolic state toward a glycolysis-dependent phenotype (8). In summary, substantial evidence has demonstrated that the remodeling of downstream metabolic enzymes and signaling pathways can drive tumor-associated metabolic imbalances in various contexts, thereby promoting cellular dysfunction and the ongoing progression of diseases through multiple mechanisms.

SWI/SNF (mSWI/SNF/BAF) is an ATP-dependent chromatin remodeling complex, with its catalytic core consisting of SMARCA4 (BRG1) or SMARCA2 (BRM). It is composed of a core scaffold/stabilizing module, which includes SMARCC1/2 (BAF155/BAF170), SMARCB1 (BAF47/INI1), SMARCE1 (BAF57), and others (9). In addition to these core components, the SWI/SNF complex also includes subunits with bridging and targeting functions, such as SMARCD1/2/3 (BAF60a/b/c), as well as a series of shared subunits, including BCL7A/B/C, ACTL6A, and β-actin. Based on the differences in subunit combinations, the SWI/SNF complex is primarily classified into three forms: canonical BAF (cBAF), characterized by subunits including ARID1A/B, DPF1/2/3, SS18, *etc.;* polybromo-associated BAF (PBAF), characterized by subunits including ARID2, PBRM1, BRD7, PHF10, *etc.;* and non-canonical BAF (ncBAF), characterized by subunits including BRD9, GLTSCR1/GLTSCR1L, etc (10). The specific subtypes of the SWI/SNF complex and their associated subunits can be found in Figure 1. These bridging/targeting subunits facilitate the precise recruitment of the SWI/SNF complex to enhancer or promoter regions through interactions with specific TFs or chromatin modification marks (such as histone modifications). Subsequently, SWI/SNF complex regulates the activity of enhancers/promoters and mediates the expression of target genes by remodeling nucleosome structures and altering DNA accessibility. Therefore, the SWI/SNF complex plays a pivotal role in chromatin plasticity and transcriptional regulation, acting as a critical epigenetic hub in metabolic regulation (11).

**Figure 1:**
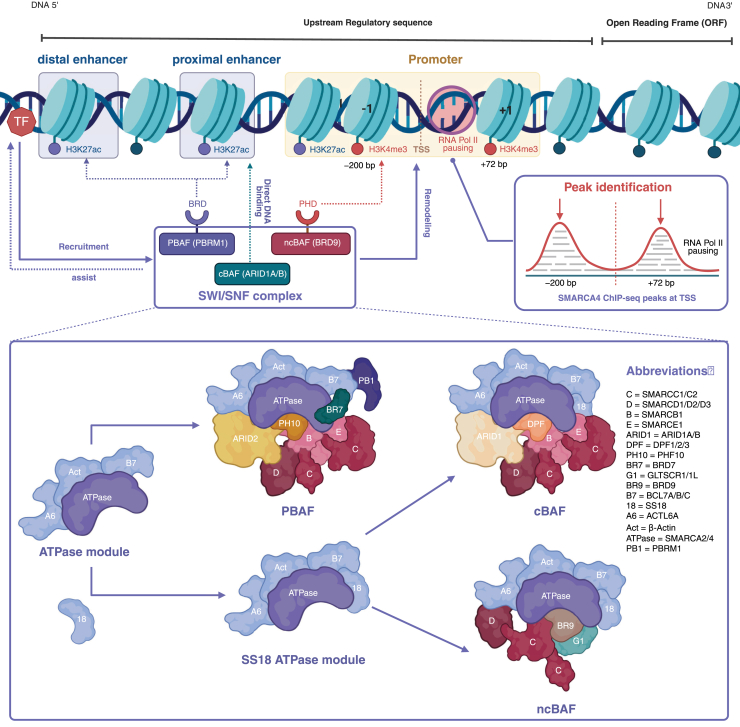
**Genome localizationand subtypes of the SWI/SNF complex**. The PBRM1 subunit in the PBAF complex contains a BRD domain that preferentially binds acetylated histones (*e.g.,* H3K27ac); the BRD9 subunit in the ncBAF complex contains a BRD domain that preferentially binds methylated histones (*e.g.,* H3K4me3). The cBAF complex, guided by its ARID1A/B subunits, binds to specific DNA sequences, thereby regulating chromatin structure and gene expression.

Recent studies combining tissue-specific genetic models and functional genomics approaches have highlighted the key regulatory role of the SWI/SNF complex in various metabolic pathways. For instance, Ming Kong and colleagues constructed liver-specific *SMARCA4* knockout mice and demonstrated that the loss of *SMARCA4* inhibits cleavage and nuclear localization of the lipid synthesis transcription factor Sterol Regulatory Element Binding Protein 1 (SREBP1), downregulating the expression of SREBP1's target genes (12). Similarly, Spaeth, J. M. and colleagues reported that in pancreatic β cells, BRG1 and BRM, catalytic subunits of the SWI/SNF complex, assemble with the transcription factor Pancreatic and Duodenal Homeobox 1 to form SWI/SNF complex, regulating islet development and insulin secretion. Knockout of these catalytic subunits results in impaired glucose tolerance (13). Furthermore, in cancer metabolism, Rugang Zhang and colleagues discovered that in ovarian clear cell carcinoma (OCCC), the loss of the cBAF characteristic subunit ARID1A induces upregulation of GLS1, driving glutamine carbon through α-ketoglutarate (α-KG) to compensate for the TCA cycle and enhance aspartate/nucleotide synthesis, creating a new metabolic dependency (14).

Therefore, this paper focuses on the role of the SWI/SNF complex in tumor metabolic reprogramming, systematically reviewing how it integrates the transcriptional regulation of metabolic genes at the chromatin level. From the perspective of epigenetic-metabolic cross-regulation, this paper provides a clearer mechanistic framework of the epigenetic regulatory network involved in tumor metabolic reprogramming.

## SWI/SNF complex in genome localization and chromatin remodeling

Multi-omics studies have demonstrated that under the guidance of cell-type-specific TFs, the SWI/SNF complex (with SMARCA4/SMARCA2 as the catalytic core) is specifically recruited to chromatin. The SWI/SNF complex exhibits two highly conserved binding patterns in the genome: first, proximal binding to promoters, where BRG1 typically localizes to the nucleosome regions at the −1 and + 1 positions flanking the transcription start site (15). This binding feature was further validated in a transcription start site meta-analysis of 501Mel melanoma cells by Laurette *et al.,* who observed BRG1 chromatin immunoprecipitation sequencing signals at −200 bp and +72 bp, coinciding with RNA polymerase II (Pol II) stalling at the +1 nucleosome on the 5′ side, forming a typical 'dual peak' configuration (16). Second, BRG1 can accumulate at both distal and proximal enhancers, with a preference for regions rich in H3K27ac. During the differentiation of embryonic stem cells to mesoderm, Alexander *et al.* observed a high overlap between BRG1 and the H3K27ac signal in distal enhancers. BRG1 loss resulted in a significant decrease in H3K27ac levels in these distal enhancers, while levels in the promoter regions remained largely unchanged, indicating that BRG1 is crucial for enhancer activation (17).

Further studies have shown that exogenous hormones or physiological stimuli can rapidly enhance the occupancy and chromatin accessibility of the SWI/SNF complex in enhancer regions, driving downstream transcriptional activation. For instance, in the β-adrenergic stimulation model in iBAT, the characteristic subunit ARID1A and its catalytic core BRG1 of the cBAF type SWI/SNF complex co-occupy the distal enhancer regions of metabolic genes such as *Adrb1* (−44 kb and −22 kb) and *Ucp1* (−13/−5/−2 kb) with Transcription factor Peroxisome Proliferator-Activated Receptor Gamma (PPARγ) and CCAAT/Enhancer Binding Protein. The binding ability is regulated by β-adrenergic agonists. After treatment with isoproterenol, the binding of JMJD1A, ARID1A, and BRG1 at the *Adrb1* and *Ucp1* enhancers was significantly enhanced. Formaldehyde-Assisted Isolation of Regulatory Elements sequencing showed an increase in chromatin accessibility at these sites, accompanied by rapid upregulation of *Adrb1* and *Ucp1* transcription (18). In addition, in the progesterone receptor activation model, José C. Reyes and colleagues found in T47D breast cancer cells that treatment with the progesterone agonist R5020 enhanced the occupancy of BAF155 at multiple progesterone receptor enhancer and mouse mammary tumor virus (MMTV) promoter regions. This was accompanied by increased DNase I sensitivity *in vitro* and the recruitment of Pol II to the enhancer regions, indicating rapid recruitment of the SWI/SNF complex upon activation, facilitating chromatin remodeling (19). Similarly, in SW-13/MMTV cells, which carry the integrated MMTV promoter and exogenously express glucocorticoid receptor and BRG1, treatment with dexamethasone induces the recruitment of GR, BRG1, and Ku70/Ku86 to the MMTV promoter region. The study demonstrates that the catalytic activity of BRG1 is critical for this process: Topoisomerase II Alpha, under its regulation, induces transient DNA double-strand breaks at the promoter region, thereby increasing the sensitivity of this region to cleavage by restriction endonucleases. Meanwhile, NucB undergoes conformational rearrangement, ultimately promoting the binding of Pol II and the initiation of hormone-dependent transcription (20). These findings highlight the crucial role of the SWI/SNF complex in regulating chromatin accessibility and enhancer activation, which is essential for transcriptional control in response to various cellular signals and stimuli.

## Co-regulation of TFs and SWI/SNF complex

One key function of the SWI/SNF complex is to drive nucleosome rearrangement through ATP hydrolysis, enhancing chromatin accessibility and creating an open chromatin environment for transcriptional activation of promoters or enhancers. However, since the SWI/SNF complex lacks clear DNA sequence specificity, its genomic localization depends on recruitment by specific TFs, and these TFs accurately target the regulatory elements of target genes.

Efficient TF binding often requires assistance from the SWI/SNF complex to remodel chromatin architecture and increase accessibility at regulatory regions of target genes. Some TFs directly interact with the SWI/SNF complex to enhance their own transactivation, while others recruit the SWI/SNF complex to remodel chromatin, supporting the binding and function of additional TFs. For example, the Caudal-related homeobox transcription factor 2 can bind BRG1 directly, recruit it to the promoters of its target genes, and thereby initiate downstream gene expression (21). Similarly, Octamer-binding transcription factor 4 recruits BRG1 to remodel nucleosome-occluded chromatin regions, which not only promotes the expression of its own target genes but also facilitates the binding of other TFs such as SRY-box transcription factor 2 and Krüppel-like factor 4. This mechanism is critical for establishing an open chromatin state during cellular reprogramming (22). Furthermore, protein arginine methyltransferase 5 interacts with Specificity Protein 1 to precisely guide BRG1 to the *Androgen Receptor (AR)* gene promoter region. During this process, protein arginine methyltransferase 5 upregulates the epigenetic mark H4R3me2s, assisting in the stable localization of BRG1, thereby enhancing the transcription of the *AR* gene (23).

**Figure 2:**
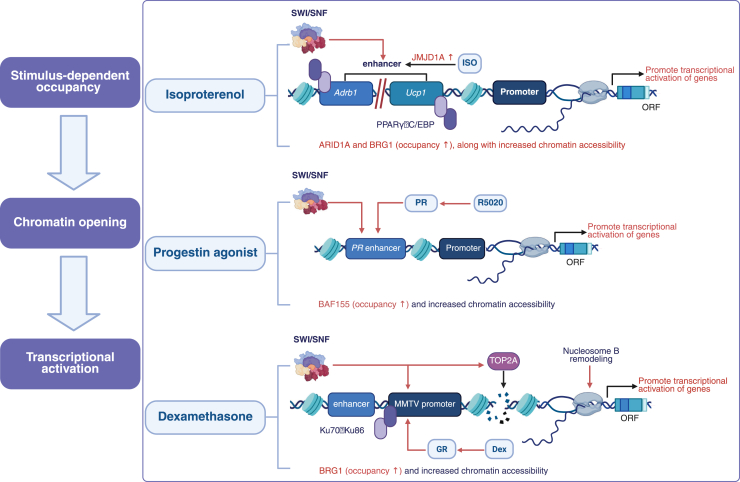
**SWI/SNF complex recruitment andchromatin remodeling in responseto external signals.**

In addition to the previously discussed mechanisms, various tissue-specific TFs interact specifically with the SWI/SNF complex, thereby guiding BRG1 to target key metabolic or disease-related genes. For example, in colorectal cancer, the Runt-related Transcription Factor 2 can mediate the targeted localization of BRG1 (24). In ovarian cancer cells, paired box 8 (PAX8) interacts with multiple SWI/SNF subunits, promoting BRG1-mediated regulation of target gene expression (25). The loss of these tissue-specific TFs, or defects in BRG1 function, lead to reduced chromatin accessibility and impaired expression of the associated genes, further highlighting the critical role of the cooperation between SWI/SNF complex and TFs in various physiological and pathological states. To provide a more intuitive representation of these mechanisms, Figure 1 illustrates the genome localization and subtypes of the SWI/SNF complex, while Figure 2 highlights its role in chromatin remodeling and emphasizes the interactions between TFs, chromatin marks, and the SWI/SNF complex in regulating enhancers and promoters in response to external stimuli.

## Regulation mechanisms of the SWI/SNF complex in carbohydrate metabolism

**Figure 3:**
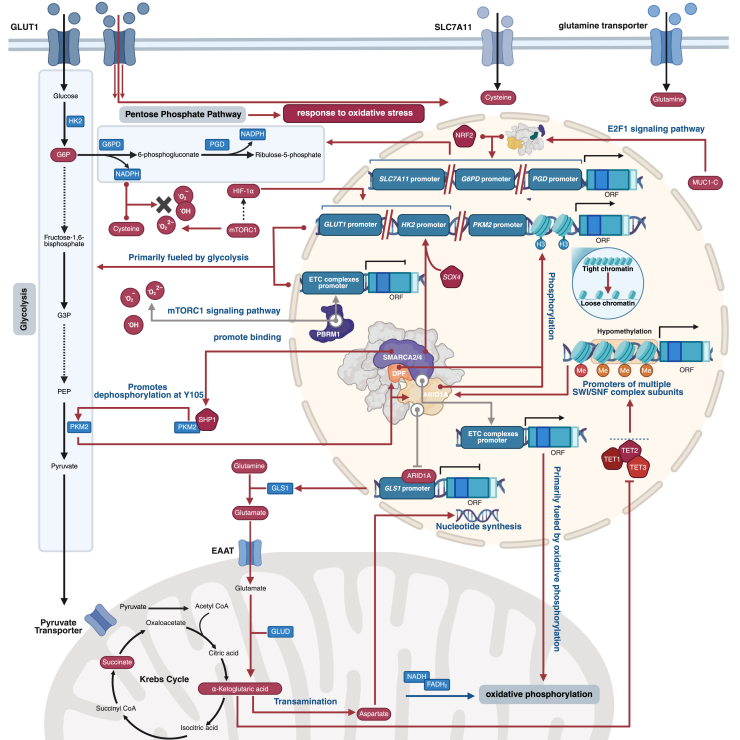
**Regulation of glucose metabolism by the SWI/SNF complex**. (PBRM1 is not shown with the complex because it is a characteristic subunit of PBAF, and displaying it alongside DPF or ARID1A from cBAF could cause misinterpretation).

Carbohydrate metabolism is the core metabolic pathway through which cells obtain energy and biosynthetic substrates. The SWI/SNF complex regulates carbohydrate metabolism through multiple mechanisms: the SWI/SNF complex can activate metabolic enzyme genes at the transcriptional level, and also remodel their conformation and subcellular localization at the post-transcriptional stage. To better understand the regulation of glucose metabolism by the SWI/SNF complex, Figure 3 illustrates how it regulates key metabolic pathways, including glycolysis, the PPP, and OXPHOS, through a complex network, while responding to various cellular signals. The SWI/SNF complex plays multiple regulatory roles in glycolysis and glycogen synthesis by modulating the expression of key metabolic genes, with functions that exhibit high diversity and context dependence (26).

### Regulatory mechanisms of the SWI/SNF complex in glycolysis

Glycolysis is a crucial step in carbohydrate metabolism, determining energy supply and carbon flow distribution in cells. Increasing evidence shows that the SWI/SNF complex is deeply involved in the regulation of glycolysis through various mechanisms, including transcriptional regulation and enzyme conformation remodeling.

#### Direct transcriptional and enzyme conformation regulation of glycolysis by the SWI/SNF complex

Pooja Khanna and colleagues confirmed through gene manipulation and functional complementation experiments that in triple-negative breast cancer (TNBC) cells, the catalytic subunit of the SWI/SNF complex, BRG1, forms a transcriptional complex with the SRY-box transcription factor 4, co-binding and directly activating the promoter of the glycolytic key enzyme HK2, thereby enhancing the conversion efficiency of glucose to G6P and increasing glycolytic flux (27). In addition, BRG1 can also interact with metabolic enzymes, such as PKM2, altering the enzymes' conformation and subcellular localization, directly impacting glycolytic activity. For example, Wenli Zhan and colleagues found that in a non-small cell lung cancer (NSCLC) model, BRG1 enhances the binding of Src homology two domain-containing phosphatase 1 to PKM2, promoting the formation of PKM2 tetramers and maintaining its cytosolic localization. The conformational change significantly increases PKM2's pyruvate kinase activity, thereby inhibiting glycolysis and shifting the tumor metabolic reprogramming toward mitochondrial OXPHOS (28).

Thus, the SWI/SNF complex regulates key nodes in the glycolysis pathway: activation of HK2 at the metabolic starting point increases glycolytic flux, while changes in PKM2 activity at the endpoint of glycolysis direct carbon flow toward mitochondria, reducing the diversion of metabolites into biosynthetic pathways, and ultimately driving the shift in metabolism from glycolysis to OXPHOS.

#### Background-dependent regulation of glycolysis: interaction mechanism of SWI/SNF subunits with tumor types

Thioredoxin Interacting Protein (TXNIP) is a key negative regulator of glucose metabolism, and its increased expression can significantly inhibit glucose uptake and glycolytic activity. However, studies show that glioma-initiating cells (GICs) lower the expression of the *TXNIP* gene through BRG1-mediated transcriptional repression, thereby maintaining glycolytic levels and overall metabolic activity. When BRG1 is deleted, the Signal Transducer and Activator of Transcription 3 (STAT3) signaling pathway is activated, with STAT3 binding to the *TXNIP* promoter, promoting transcription, upregulating TXNIP expression. Increased TXNIP expression further inhibits the expression of glycolysis-related genes such as *GLUT1, FBP1,* and *PKM,* ultimately weakening the cell's ability to utilize glucose (29).

Similar mechanisms are observed in other tumors such as muscle-invasive bladder cancer and clear cell renal cell carcinoma (ccRCC). A clinical study on advanced muscle-invasive bladder cancer found that the expression of core SWI/SNF scaffold subunits (such as SMARCA2, SMARCB1, and SMARCC1) is closely related to key factors of glucose metabolism (PKM2, AMP-activated protein kinase alpha 1. Both are often found to be co-dysregulated in tumor tissues, and the co-dysregulation of glucose metabolism-related factors is highly correlated with poor patient prognosis (including metastasis, recurrence, and survival). Additionally, BRM's regulation of glucose metabolism shows a clear cell subtype dependence. In the T24 cell line, which has high glycolytic activity, overexpression of BRM significantly upregulates various glycolysis-related genes (such as *PKM2, HK, LDHA, Aldolase A*), thereby enhancing glycolytic flux. Meanwhile, LDHA is downregulated, suggesting that the cells cannot effectively adjust metabolism to adapt to changes in energy levels. In contrast, in the 5637 cell line, a human urinary bladder carcinoma cell line, which has low metabolic activity, overexpression of BRM has no significant effect on the activation of glycolysis-related genes but instead upregulates the expression of the *FBP1* gene, suggesting that BRM may inhibit glycolysis and enhance metabolic homeostasis regulation in this context (30).

Basudev Chowdhury and colleagues further investigated this in a ccRCC model that re-expression of BAF180 in Caki2 cells, which lack PBAF, significantly upregulates several hypoxia response-related genes (such as *IGFBP1, PHD3,* and other HIF1α target genes), and modulates the expression and function of HIF1α. These findings suggest that BAF180 may mediate metabolic reprogramming by regulating HIF1α-related pathways. However, reexpression of BAF180 also inhibits the activation of the Phosphoinositide 3-Kinase/Protein Kinase B signaling pathway, thereby reducing the expression of GLUT1 and VEGF, both of which in turn suppress glycolysis and angiogenesis, weakening the metabolic adaptability of tumor cells (31).

Similarly, in lung adenocarcinoma, loss of the cBAF-specific subunit ARID1A significantly increases chromatin accessibility, particularly in the promoter regions of key glycolytic genes such as *pgam1, Pkm,* and *Pgk1,* genes that become more open. The increased chromatin accessibility enhances HIF1α enrichment in these regions, reduces recruitment of Histone Deacetylase 1, and increases H4 acetylation, promoting BRD4 binding and activating glycolytic gene transcription. Ultimately, this drives tumor metabolic reprogramming and progression (32).

These studies highlight the complexity of SWI/SNF subunit-mediated regulation of glycolysis, illustrating the role of differential expression and recruitment of specific subunits in various tumor types drive metabolic reprogramming and influence tumor progression.

#### Metabolic compensation mechanisms induced by SWI/SNF subunit deletion

The above findings suggest that, although the core or structural subunits of the SWI/SNF complex may inhibit glycolysis in certain contexts, tumor cells still utilize metabolic compensation mechanisms to maintain energy homeostasis in order to support continuous growth and proliferation. Studies have shown that in the context of *SMARCA4* deletion, the transcription of glycolysis-related genes (such as *GLUT1, HK2,* phosphofructokinase 2/fructose-2,6-bisphosphatase 3, and *PKM2*) is significantly suppressed. Further metabolic flux analysis revealed that, while these tumor cells exhibited a decreased glycolytic capacity, the tumor cells displayed higher Oxygen Consumption Rate and lower Extracellular Acidification Rate, suggesting that their metabolic reprogramming shifted towards mitochondrial OXPHOS (33).

To further elucidate the metabolic reprogramming mechanisms in the context of SWI/SNF complex subunit deletion, the Sidong Huang *et al.* utilized the DepMap genome-wide CRISPR knockout database to identify metabolic genes and pathways that are specifically dependent on the *SMARCA4*/*SMARCA2* double knockout cells. The study revealed that the double deletion of *SMARCA4* and *SMARCA2* significantly impaired the cells' glycolytic capacity and glycolytic reserve, particularly downregulating the gene *SLC2A1,* which encodes GLUT1. The downregulation of SLC2A1 implies a limitation in glucose uptake and glycolytic flux. Metabolomic and ^13^C isotope tracing experiments further confirmed that, as glucose utilization decreased, glutamine flux significantly increased, indicating that the cells' metabolic reprogramming shifted to rely more on glutamine to sustain energy supply (34). Specifically, *SMARCA4*/*SMARCA2* double knockout cells enhance glutamine uptake and utilization efficiency by upregulating *SLC38A2,* prioritizing the conversion of glutamine's carbon skeleton into glutamate and α-KG. These metabolites serve as key substrates for the TCA cycle, sustaining OXPHOS activity and compensating for the energy and biosynthetic substrate gaps caused by the limitation in glycolysis.

The above studies suggest that metabolic compensation mechanisms induced by the *SMARCA4*/*SMARCA2* double knockout can be targeted through several intervention strategies. First, the GLS1 inhibitor CB-839 can significantly block the carbon flux from glutamine into the TCA cycle, weakening the energy dependence of mitochondrial OXPHOS (35). Secondly, enhanced dependence is another metabolic feature of tumors with *SMARCA4*/*SMARCA2* loss. The mitochondrial complex I inhibitor IACS-010759 enhanced metabolic dependence by inducing energy depletion and aspartate deficiency, inhibiting tumor growth (36). Additionally, SLC38A2, as a major glutamine transporter, also mediates alanine transport. Studies have shown that exogenous supplementation of alanine can competitively inhibit SLC38A2 activity, which limits glutamine uptake. This can induce a metabolic crisis and cell death in double knockout cells, offering potential therapeutic value (34).

These findings suggest that the SWI/SNF complex does not have a uniform, unidirectional role in tumor metabolic regulation. Its function is far from being limited to promoting glycolysis or supporting tumor proliferation. On the contrary, the SWI/SNF complex's regulatory effects are significantly subtype-dependent and metabolism-context-specific. In different tumor subtypes or cellular metabolic states, it may exhibit completely different or even opposing regulatory patterns.

It is important to note that although the loss of SWI/SNF subunits may suppress glycolysis in certain contexts, such suppression of glycolysis does not imply a decrease in overall tumor metabolic activity. On the contrary, tumor cells can activate alternative pathways through metabolic reprogramming, such as enhancing SLC38A2-mediated glutamine uptake, to sustain TCA cycle and OXPHOS activity, thus meeting the demands for energy and biosynthetic substrates. This metabolic compensation mechanism highlights the SWI/SNF complex in maintaining tumor metabolic homeostasis.

#### Feedback regulation of glycolytic enzymes on SWI/SNF complex

In addition to its positive regulation of glucose metabolism by the SWI/SNF complex, recent studies have also shown that core glycolytic enzymes can reciprocally regulate the assembly and function of SWI/SNF complex. For instance, the glycolytic enzyme PKM2 upregulates the expression of key SWI/SNF subunits, DPF2 and ARID1A, enhancing their chromatin binding at the promoters of myogenic genes (*e.g., myogenin, MyhCIIb*), thereby activating the transcription of differentiation-related genes. Moreover, PKM2 directly promotes the phosphorylation of H3T11, and, by regulating nuclear PKC and AKT kinases, indirectly enhances the phosphorylation of H3T6 and H3T45. The modifications of H3T11, H3T6, and H3T45 work together to improve chromatin structure and promote the activation of myogenic genes (37). In contrast, PKM1 primarily promotes the nuclear localization of DPF2, facilitating its chromatin binding, and complements PKM2 in function (37).

### Metabolic interaction mechanism between SWI/SNF complex and the TCA

The TCA cycle, as the core metabolic pathway for further oxidation of glycolytic products, drives OXPHOS through the generation of reducing equivalents (NADH, FADH_2_) and provides energy and key intermediate metabolites to maintain metabolic homeostasis. Although systematic studies directly addressing the regulation of key TCA enzymes (*e.g.,* Pyruvate Dehydrogenase, Citrate Synthase) by the SWI/SNF complex are currently lacking, existing research suggests that, under specific subunit deletion contexts, SWI/SNF complex may indirectly affect TCA activity by regulating glutamine metabolism. However, whether the SWI/SNF complex is involved in the transcriptional or functional regulation of TCA enzymes remains unclear and requires further investigation.

In this context, GLS1 is a key enzyme catalyzing the conversion of glutamine to glutamate. The structural subunit of the SWI/SNF complex, ARID1A, can bind to the *GLS1* promoter and inhibit its transcriptional activity. However, in OCCC, the deletion of *ARID1A* relieves the inhibition, leading to a significant upregulation of GLS1 protein and enhanced glutamine metabolism. Upregulated glutamate is then converted into α-KG by enzymes like glutamate dehydrogenase, entering the TCA to support mitochondrial metabolism. ^13^C_5_-glutamine tracing experiments show that *ARID1A* deletion significantly promotes the accumulation of TCA intermediates such as citrate and malate and increases the synthesis level of aspartate (14). Notably, aspartate, as an important intermediate connecting the TCA cycle with nucleotide synthesis, is elevated in *ARID1A*-deficient cells, which helps meet the metabolic demands for rapid DNA/RNA synthesis in tumor cells (38).

Building on this understanding, a study on the significant elevation of α-KG levels in the urine of interstitial cystitis patients revealed its potential epigenetic regulatory role on the SWI/SNF complex. Mechanistically, α-KG competitively inhibits the activity of the Ten-Eleven Translocation family of demethylases, blocking DNA demethylation processes. α-KG inhibition leads to hypomethylation of the promoter regions of several SWI/SNF subunits, including ARID1A and SMARCA2, accompanied by their transcriptional upregulation. Ultimately, this significantly inhibits the abnormal proliferation of bladder epithelial cells (39). A similar mechanism has also been observed in case studies of *Succinate Dehydrogenase Complex Subunit B*-deficient renal cell carcinoma. Immunohistochemistry shows that in this type of tumor, FBP1 expression is downregulated, while PKM2 is upregulated, indicating a metabolic profile with enhanced glycolysis and impaired TCA function. *Succinate Dehydrogenase Complex Subunit B* deficiency leads to significant accumulation of succinate in the cells, which structurally resembles α-KG. Researchers speculate that succinate may inhibit α-KG-dependent demethylases through a mechanism similar to α-KG, thereby upregulating the expression of SWI/SNF subunits such as SMARCA4, SMARCB1, and PBRM1 (40). It is important to note that while the above studies establish an epigenetic link between metabolic products and gene expression, much of the current focus is on the upstream regulation of SWI/SNF complex by metabolites. The underlying mechanisms remain incomplete, and systematic studies on whether SWI/SNF complex can reverse-regulate the expression of TCA key enzymes or overall metabolic flux are still lacking.

### Functional differences in the regulation of OXPHOS and electron transport chain by SWI/SNF subunit loss

Gene expression profiling of *PBRM1*-mutant samples in ccRCC revealed that the loss of *PBRM1* activates the mTORC1 signaling pathway. On one hand, mTORC1 activation leads to an increase in intracellular reactive oxygen species (ROS) levels, which exacerbates mitochondrial dysfunction (41). On the other hand, mTORC1 activation promotes the expression of glycolysis-related genes, such as *GLUT1* and *HK2,* enhancing glucose uptake and metabolic capacity (42). The combination of these two effects forces tumor cells to undergo metabolic reprogramming, shifting their energy production from OXPHOS to predominantly glycolysis. Meanwhile, mTORC1 activation also regulates mitophagy (43), which can be impaired under increased ROS conditions. As a result, mitochondrial damage is further aggravated. These changes establish a negative feedback loop in the regulation of OXPHOS, further weakening mitochondrial function and increasing the tumor cells' reliance on glycolysis.

In contrast, the loss of *SMARCA4* enhances OXPHOS activity by upregulating mitochondrial respiration-related genes and key metabolites in the TCA cycle, such as glutamine and α-KG, suggesting a shift toward mitochondrial oxidative metabolism (44). The metabolic shift toward mitochondrial oxidative metabolism is further supported by the upregulation of Peroxisome proliferator-activated receptor gamma coactivator 1-alpha (PGC-1α), which promotes mitochondrial biogenesis and enhances OXPHOS function, thus supporting tumor proliferation (45).

Similarly, the loss of *ARID1A* increases the expression of electron transport chain (ETC)-related genes, improving electron transfer efficiency and ATP synthesis capacity, while also promoting mitochondrial fission through the upregulation of c-Myc, and this further increases mitochondrial number and activity in OCCC cells (46, 47).

The previous findings reveal the distinct effects of SWI/SNF subunit loss on mitochondrial function, with *PBRM1* loss promoting a shift from OXPHOS to glycolysis, while the loss of *SMARCA4* and *ARID1A* enhances OXPHOS activity and ETC-related gene expression, supporting tumor metabolism and progression.

### Regulation of the PPP by SWI/SNF complex

In addition to regulating OXPHOS, the SWI/SNF complex can also regulate the PPP, significantly enhancing the antioxidant stress capacity of tumor cells, revealing its multiple roles in maintaining metabolic adaptability.

Recent studies have shown, research has shown that in prostate cancer (PCa), the oncogene MUC1-C (Mucin 1, C-terminal subunit) can activate the E2F1 signaling pathway, leading to the upregulation of specific subunits of the PBAF complex, such as PBRM1, ARID2, and BRD7. The activation of the PBAF complex enhances chromatin remodeling and facilitates Nuclear factor erythroid 2-related factor 2 (NRF2) recruitment to its target gene promoters. As a result, the activation of the PBAF complex and NRF2 pathway works synergistically to enhance the expression of downstream antioxidant genes, including *SLC7A11, G6PD,* and *PGD.* This mechanism drives the flux through the PPP, increases NADPH production, and enhances the cell's capacity to scavenge ROS, thereby maintaining metabolic homeostasis (48). These findings emphasize the critical role of MUC1-C in orchestrating PBAF and NRF2 pathways to protect cells from oxidative stress and support cancer cell survival. Therefore, the SWI/SNF complex, especially PBAF, enhances cancer cell metabolic adaptability and resistance to radiotherapy and chemotherapy by regulating the PPP pathway, promoting PCa progression and drug resistance (48). Moreover, these findings are highly consistent with those of Ye Lv and colleagues, further validating the key role of the SWI/SNF complex in metabolic adaptation in PCa cells. Specifically, under androgen deprivation and oxidative stress conditions, BRD9 undergoes S-glutathionylation (the modification of proteins by glutathione), which enhances its binding capacity with Nuclear Factor Y Subunit A (NFYA). The BRD9–NFYA complex synergistically activates the expression of Glycogen Phosphorylase L, promoting glycogenolysis and redirecting glucose flux into the PPP, thereby increasing G6PD activity, enhancing NADPH production, improving ROS scavenging ability, and maintaining cellular redox homeostasis (49).

Additionally, the study also points out that the BRD9–NFYA axis promotes glycogenolysis by inducing Phosphorylase L expression. The glucose released from the glycogenolysis process not only enhances glycolytic flux but is also preferentially diverted to the PPP, rather than entering mitochondrial oxidative metabolism. This mechanism enhances NADPH production and ROS scavenging ability, thereby boosting PCa cell adaptability to oxidative stress under androgen deprivation conditions. Furthermore, the study underscores the crucial role of the PBAF subunit in regulating the PPP, which enhances cancer cell metabolic flexibility, supports antioxidant defenses, and promotes tumor progression and resistance to therapy.

## Regulatory mechanism of SWI/SNF complex in lipid metabolism

Lipid metabolism is a complex and dynamic process involving multiple steps, including fatty acid synthesis and oxidation. Recent studies have shown that the SWI/SNF complex plays a central role in regulating lipid metabolism through interactions with various TFs and metabolic signaling pathways.

### The role of the SWI/SNF complex in the regulation of fatty acid synthesis

The SWI/SNF complex serves as a crucial cofactor for PPARγ-mediated processes such as adipogenesis, lipid metabolism, thermogenesis, and glucose homeostasis, playing an important regulatory role in fatty acid synthesis pathways (50). Specifically, the SWI/SNF complex subunit SMARCA4 exhibits significant transcriptional regulatory function by modulating the expression of genes involved in fatty acid synthesis as part of the SWI/SNF complex. In the TNBC cell line MDA-MB-231, ChIP-qPCR experiments revealed that BRG1 binds to the promoter regions of key lipid synthesis enzymes, such as *Acetyl-CoA Carboxylase (ACC), Fatty Acid Synthase (FASN), ATP Citrate Lyase* (*ACLY*), *Acyl-CoA Synthetase Long-Chain Family Member 1*, and the intronic active region of *LPIN1* (51). Additionally, BRG1 is involved in the regulation of lipid metabolism by interacting with Sterol Regulatory Element-Binding Protein 1c. BRG1 and Sterol Regulatory Element-Binding Protein 1c are recruited to the promoter regions of target genes, enhancing the transcription of lipid metabolism-related genes and promoting fatty acid synthesis (52, 53). Furthermore, in hepatocellular carcinoma (HCC), BRG1 also binds to and represses the *Glia Maturation Factor Beta* promoter, regulating the PIK3AP1/ Phosphoinositide 3-Kinase/Protein Kinase B signaling pathway to promote lipid droplet accumulation (54).

Notably, under *SMARCA4* deletion, its regulatory effects on fatty acid synthesis may exhibit drastically different directionalities. For instance, in NSCLC, *SMARCA4* deletion results in a significant decrease in phosphorylated pACC-Ser79 levels. As this site is the key phosphorylation site for ACC inhibition by AMPK, a reduction in its level suggests enhanced ACC activity and accelerated fatty acid synthesis. Meanwhile, *SMARCA4* deletion is also accompanied by a decline in glycolytic capacity, and this further exacerbates the cell's reliance on mitochondrial OXPHOS. This "energy supply limitation, increased energy consumption associated with anabolic metabolism" state leads to energy imbalance and restricts tumor cell growth. The application of fatty acid synthesis inhibitors (such as Cpd-10v or IPI-9119) or the mTOR inhibitor rapamycin can reduce energy consumption associated with anabolic metabolism, partially alleviate the inhibitory effect of IACS-010759 on cell proliferation, and relieve cellular energy stress (45). Similarly, in liver models, the deletion of *ARID1A* leads to significant upregulation of fatty acid synthesis-related genes (such as *FASN, Stearoyl-CoA Desaturase 1, Acyl-CoA Synthetase Short-Chain Family Member 2*), while FAO-related genes (such as *CPT1A, CPT2, Acyl-CoA Synthetase Long-Chain Family Member 1*, *ACSM1, ACSF2*) are significantly downregulated (55).

These findings indicate different subunits of the SWI/SNF complex (such as ARID1A, SMARCA4) play distinct roles in lipid metabolism. The same subunit may exhibit different or even opposing metabolic regulatory functions depending on the tissue environment or the specific SWI/SNF subcomplex involved.

### The role of SWI/SNF complex in regulating fatty acid β-oxidation

The SWI/SNF complex also plays a key regulatory role in fatty acid β-oxidation. Among its subunits, BAF60a not only induces the expression of fatty acid β-oxidation-related genes (*e.g., Hadha* and *Acaa2*) but also interacts with the transcriptional coactivator PGC-1α, suggesting BAF60a′s role in promoting FAO in liver metabolism (56). Furthermore, research by Laura Moody and colleagues found that treatment with non-esterified fatty acids significantly upregulates the transcript abundance of *Cpt1a* in hepatocytes. Further analysis revealed that the binding of PPARα and CCAAT/Enhancer Binding Protein β to the regions of the *Cpt1a* promoter was significantly enhanced, accompanied by enhanced enrichment of BAF60a and Pol II. The results suggest that the SWI/SNF complex may be recruited to the *Cpt1a* gene region through a BAF60a-mediated mechanism, working synergistically with lipid metabolism-related TFs to promote transcriptional activation of the gene. As a result, the transcript abundance of Cpt1a is upregulated, leading to increased CPT1A protein abundance, mitochondrial activity is enhanced, and the liver's fatty acid β-oxidation capacity increases (57).

### Nutrient-status-dependent regulation of lipid metabolism by the SWI/SNF complex

Further research has shown that the subunit BAF60a of the SWI/SNF complex acts as a nutrient status-dependent co-regulator, dynamically modulating metabolic pathways through selective binding with different TFs. Specifically, during fasting, BAF60a interacts with the transcriptional coactivator PGC-1α, synergistically activating the expression of genes related to FAO, thereby enhancing mitochondrial oxidative metabolism. In contrast, under overnutrition conditions, such as high-fat diets or excess non-esterified fatty acids, BAF60a forms a complex with Y-box binding protein 1, inhibiting the transcriptional activity of urea cycle-related genes (such as *Cps1* and *Otc*), shifting metabolic flow from nitrogen metabolism to lipid metabolic pathways (58).

This "FAO–urea cycle" linkage regulatory mechanism suggests that the SWI/SNF complex, especially its subunit BAF60a, can dynamically redistribute metabolic flux between carbon and nitrogen metabolism in response to changes in the nutritional environment, thereby coordinating the regulation of energy and nitrogen homeostasis in the body.

## The bridging role of amino acids in carbon-nitrogen metabolic coupling

**Figure 4:**
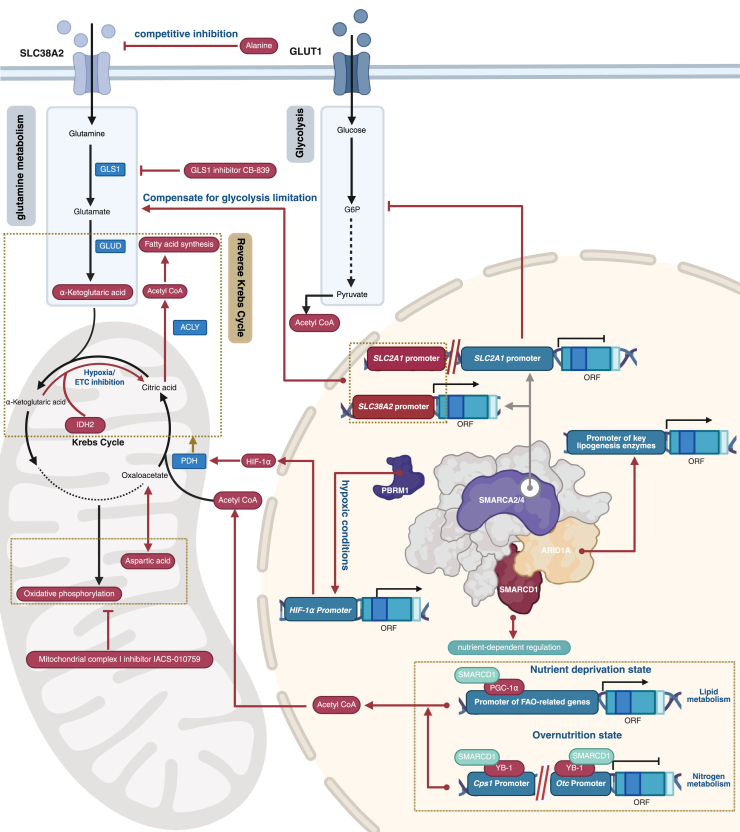
**The SWI/SNF complex regulation of metabolic compensation and carbon-nitrogen flux coupling**. (PBRM1 is not shown with the complex because it is a characteristic subunit of PBAF, and displaying it alongside ARID1A from cBAF could cause misinterpretation).

As discussed earlier, glutamine provides energy compensation through the TCA and OXPHOS pathways. At the same time, BAF60a, a bridging subunit of the SWI/SNF complex, acts as a nutrient-sensing co-regulator, enabling BAF60a to facilitate a programmed metabolic switch between FAO and urea synthesis. Figure 4 illustrates the regulatory mechanisms of the SWI/SNF complex in metabolic compensation and carbon-nitrogen flux coupling, offering a clearer understanding of the interrelationship between these processes.

Recent studies have also revealed that under hypoxic conditions or when the mitochondrial respiratory chain is impaired (such as ETC inhibition), cells can utilize a reductive carboxylation pathway mediated by IDH2 to convert glutamine into carbon sources necessary for lipid synthesis. In this pathway, α-KG is reversibly reduced to citrate, which is then cleaved by ACLY into acetyl-CoA, entering the lipid synthesis pathway (59). There is no direct evidence suggesting the SWI/SNF complex regulates the reductive carboxylation pathway. Several studies indicate that its subunits may indirectly influence this metabolic reprogramming process through upstream and downstream mechanisms. Firstly, PBRM1 (a PBAF-specific subunit) can regulate the expression of HIF-1α target genes, and these target genes in turn inhibit Pyruvate Dehydrogenase activity indirectly by upregulating PDK1 and PDK3, and enhance the reverse metabolism of α-KG into citrate (60, 61), promoting the diversion of glutamine-derived carbon for lipid synthesis (62). Secondly, SWI/SNF subunits such as SMARCA4 and ARID1A can regulate the expression of key lipid synthesis enzymes such as ACLY, FASN, and *Acyl-CoA Synthetase Short-Chain Family Member 2*. In the activated lipid synthesis state, the cellular demand for citrate and acetyl-CoA increases, which may further promote the flux of glutamine through the IDH1/2-mediated carboxylation pathway, providing a carbon source for lipid synthesis (51). Therefore, although the SWI/SNF complex has not been directly confirmed to regulate the IDH1/2-mediated glutamine carboxylation, its roles in hypoxic response and lipid metabolism regulation provide biological plausibility for its potential indirect regulatory function.

## SWI/SNF complex and tumor metabolic reprogramming

The SWI/SNF complex is crucial for regulating gene transcription and maintaining cell lineage stability. Mutations or functional loss of its core subunits (such as SMARCC1, SMARCA4, SMARCB1, *etc.*) frequently occur in various cancers. Recent advances in tumor metabolism research have highlighted the key role of the SWI/SNF complex in regulating cell energy balance, proliferation, and metabolic adaptation. The SWI/SNF complex not only drives ATP-dependent chromatin remodeling but also collaborates with various TFs to regulate critical metabolic processes, such as glycolysis, mitochondrial respiration, lipid peroxidation, and ferroptosis (63). These findings suggest that SWI/SNF complex abnormalities may serve as an important entry point for metabolic-targeted therapies.

To provide a clearer representation of the relationship between SWI/SNF complex and tumor metabolism, Table 1 summarizes the metabolic pathways and regulatory mechanisms involved in current relevant studies. The following content will focus on three tumor types—ccRCC, NSCLC, and HCC—which have been studied more extensively, and will elaborate on their details.

**Table 1:** Impact of SWI/SNF subunit mutations on metabolic pathways in various tumor types

Tumor type	SWI/SNF subunit alterations	Major Affected metabolic Pathways/Nodes	Functional effects
Clear Cell Renal Cell Carcinoma	PBRM1	ETC/OXPHOS; Glycolysis; HIF Signaling	Enhanced HIF signaling upon *PBRM1* loss; Downregulation of ETC/OXPHOS genes; Upregulation of glycolysis/angiogenesis programs.
	SMARCA4	OXPHOS dependency; Mitochondrial respiration (ETC complexes I–IV)	Induces a targetable dependence on OXPHOS; Increased mitochondrial respiratory activity and OXPHOS reliance.
Non-Small Cell Lung Cancer	SMARCA4	Terminal glycolysis (PKM2-Y105); OXPHOS; Lipid peroxidation; Ferroptosis-associated redox metabolism	Promotes PKM2 dephosphorylation and tetramerization; Suppresses glycolysis; Favors OXPHOS; Leads to altered lipid peroxide clearance and ferroptosis sensitivity.
Hepatocellular Carcinoma	ARID1A	Glycolysis; TCA/OXPHOS; Cuproptosis	Lowers PKM/glycolysis and shifts reliance to TCA/OXPHOS, conferring hypersensitivity to TCA-targeted cuproptosis.
	SMARCA4	Lipid peroxide clearance; Antioxidant metabolism	Transcriptionally activates 4-hydroxynonenal detoxifying enzymes, enhancing ROS adaptability and survival.
Ovarian Clear Cell Carcinoma	ARID1A	Glutamine metabolism; GLS1; TCA anaplerosis; OXPHOS	Directly represses GLS1; its loss creates glutamine/TCA-OXPHOS dependence.
Triple-Negative Breast Cancer	SMARCA4	Glycolysis initiation (HK2); *de novo* lipogenesis (*Acetyl-CoA Carboxylase* / *Fatty Acid Synthase* / *ATP Citrate Lyase*); SREBP-driven lipid programs	Forms a complex with SRY-box transcription factor 4 to activate the *HK2* promoter, increasing glycolytic flux; BRG1 enhances the transcription of lipogenic genes, promoting lipogenic programs and increasing dependence on fatty acid synthesis.
Prostate Cancer	BRD9	Glycogenolysis; PPP; NADPH metabolism	BRD9–NFYA induces Phosphorylase L, diverting carbon to PPP and boosting NADPH/ROS clearance.
	PBRM1	PPP; Antioxidant metabolism	MUC1-C–E2F1 upregulates PBAF components that cooperate with NRF2 to enhance PPP/antioxidant programs and maintain redox homeostasis.
Glioma-Initiating Cells	SMARCA4	TXNIP–glucose-uptake inhibitory axis; Glycolytic gene program	Represses TXNIP to sustain glycolysis; Loss activates Signal Transducer and Activator of Transcription 3, elevates *TXNIP,* suppresses glycolytic genes (*e.g., SLC2A1*/*GLUT1, FBP1, PKM*), and lowers glucose utilization.
Lung Adenocarcinoma	ARID1A	Glycolysis (*e.g.,* PGAM1, PKM, PGK1); HIF1α–BRD4 axis	Loss increases promoter accessibility of glycolytic genes and enhances HIF1α/BRD4 binding, promoting a glycolytic phenotype.
Renal Medullary Carcinoma	SMARCB1	TFCP2L1–MYC program; NRF2-linked redox programs; lipid peroxidation and the ferroptosis axis	Activation of *T*FCP2L1–MYC and NRF2 programs upon *SMARCB1* loss; Ferroptosis resistance; Reversal of programs and induction of ferroptotic death upon *SMARCB1* re-expression.
Small Cell Carcinoma of the Ovary, Hypercalcemic Type	SMARCA4	Nucleotide metabolism and folate-mediated purine synthesis; OXPHOS dependency	Loss increases reliance on one-carbon and nucleotide synthesis; Sensitivity to methotrexate and DHODH inhibitors

OXPHOS, oxidative phosphorylation; NRF2, nuclear factor erythroid 2-related factor 2; TXNIP, thioredoxin interacting protein.

### SWI/SNF complex and ccRCC

In ccRCC, *VHL* gene inactivation is the most common molecular feature. In a 2011 study by Ignacio Varela *et al.,* PCR exon resequencing of approximately 3500 genes identified *PBRM1* as the second most commonly mutated gene in ccRCC, after *VHL,* with a mutation rate of about 40% (64). Subsequently, other subunits of the SWI/SNF complex, such as *SMARCA2* and *SMARCB1,* were also found to have functional loss, suggesting that these subunits play an important role in the initiation and progression of renal cancer (41).

In renal proximal tubular cells, PAX8 is a key TF that maintains epithelial lineage stability and differentiation programs, and its normal transcriptional activity depends on the PBAF complex, which includes *PBRM1.* After *PBRM1* deletion, PAX8 fails to recruit PBAF effectively and instead associates with co-repressors like DNMT1, NuRD, NCoR/SMRT, SIN3A, and ISWI, shifting its role from activation to repression. This results in significant downregulation of several downstream genes that regulate epithelial differentiation, such as *GATA binding protein 3* , *LIM homeobox transcription factor 1*, and *Wilms' tumor 1,* impairing the differentiation program and causing the cells to remain in an undifferentiated state. This lineage suppression is prominent in *Pbrm1* knockout mice, supporting its role in ccRCC pathogenesis (65).

*PBRM1* is a typical early clonal mutation in tumorigenesis, persisting throughout cancer evolution (66). In the context of *VHL* deletion, *PBRM1* mutation is associated with enhanced transcriptional programs dependent on HIF1α and STAT3, accompanied by downregulation of mitochondrial metabolic programs (including reduced expression of *OXPHOS* gene clusters for Complexes I, II, IV, and V) and upregulation of glycolysis and angiogenesis genes. At the same time, mTORC1 signaling remains persistently activated, with a decrease in the expression of upstream inhibitory factors TSC1/TSC2, and increased phosphorylation of downstream effectors eukaryotic translation initiation factor 4E-binding protein 1 and ribosomal protein S6 kinase. These changes highlight the cooperative mechanisms between *VHL* inactivation and *PBRM1* loss in transcriptional regulation, metabolic reprogramming, and growth signal activation (67).

Although BAF47 and BRM have not yet been directly confirmed to participate in metabolic reprogramming in ccRCC, as key subunits of the SWI/SNF complex, these subunits play important roles in maintaining renal lineage stability, epithelial differentiation, and restricting tumor cell plasticity (68). *SMARCB1* loss can induce chromatin state remodeling, promoting stem cell-like epigenetic features, while BRM exhibits a typical tumor suppressor function, with its low expression being closely associated with enhanced proliferation, migration, and invasion of ccRCC cells. Although these changes are not directly involved in metabolic pathways, they may indirectly influence metabolic plasticity and regulatory potential through lineage state or structural remodeling, providing clues for further research (69, 70).

### SWI/SNF complex and NSCLC

BRG1, a SWI/SNF subunit encoded by *SMARCA4,* is a commonly mutated or downregulated gene in NSCLC, and its loss is closely associated with poor prognosis and chemoresistance. Studies have shown that BRG1 is generally downregulated in lung cancer tissues; this downregulation results in, as mentioned earlier, a shift in metabolism from glycolysis dependence to mitochondrial oxidation, leading to increased dependence on mitochondrial OXPHOS in NSCLC with *SMARCA4* deletion. Blazanin *et al.* discovered that OXPHOS inhibitors could induce tumor cells to upregulate glycolysis to compensate for energy demands. Further CRISPR screening identified ROCK1/2 as key cooperative targets, and the ROCK inhibitor KD025 could block this compensatory glycolytic response, exacerbating the energy crisis and inducing cell cycle arrest. This reveals a synthetic lethal strategy involving 'OXPHOS dependence + glycolysis compensation (71).

In another study, CRISPR/Cas9 screening showed that *SMARCA4* deletion significantly inhibits ferroptosis. Through transcriptomic and epigenomic analyzes, *Aldehyde Dehydrogenase 16 Family Member A1 (ALDH16A1)* was identified as a direct target gene of BRG1, and its pro-ferroptosis function is independent of aldehyde dehydrogenase activity. Mechanistically, BRG1 upregulates ALDH16A1 expression by enhancing the chromatin accessibility of its promoter; ALDH16A1 subsequently activates the lysosomal degradation pathway, reducing the stability of thioredoxin (TXN) protein, and binds to the TXN active site to inhibit its redox function. These changes result in decreased lipid peroxidation clearance, thereby increasing the cell's sensitivity to ferroptosis. Unlike classical GPX4/GSH or FSP1/CoQ10-dependent antioxidant mechanisms, the metabolic adaptation induced by *SMARCA4* deletion represents a TXN system-dominant "anti-ferroptosis metabolic barrier (72)."

### SWI/SNF complex and hepatocellular carcinoma

In HCC, functional defects of SWI/SNF subunits are closely associated with metabolic reprogramming. *ARID1A* mutations or deletions, found in approximately 10% of HCC cases, are one of the critical defects of the SWI/SNF complex (73).

Studies show that *ARID1A* deletion weakens glycolytic capacity, forcing tumor cells to rely on mitochondrial OXPHOS and the TCA cycle to maintain energy supply. This deletion maintains the accessibility and transcriptional activity of key glycolytic enzyme promoters. When deleted, PKM expression and enzymatic activity decrease, leading to reduced glycolysis and pyruvate production, which induces compensatory enhancement of mitochondrial respiration. Notably, cuproptosis, a programmed cell death pathway dependent on mitochondrial TCA cycle and OXPHOS activity, exhibits significant synthetic lethality sensitivity in *ARID1A*-deficient HCC (*e.g.,* elesclomol/Cu^2+^ treatment) (74). Recent studies also found that *ARID1A* deletion activates the AMPK stress pathway, enhancing cell survival under glucose-deprived conditions. This suggests that AMPK inhibitors may represent a potential therapeutic strategy (75).

Another catalytic subunit, BRG1, also promotes HCC survival by regulating redox metabolism. Immunohistochemical analysis of 221 HCC tissue samples showed that the level of the lipid peroxidation byproduct 4-hydroxynonenal (4-HNE) was generally low, and low levels of 4-HNE were associated with poor prognosis. Further validation through database predictions and functional experiments confirmed that BRG1 regulates the transcription of 4-HNE clearance-related enzymes such as GSTA4, AKR, and ALDH, accelerating the metabolism and clearance of 4-HNE. This enhances the tumor cells' adaptation to oxidative stress, promoting the progression of HCC (76).

## Discussion

This review systematically summarizes the multifaceted regulatory roles of the SWI/SNF complex in tumor metabolic reprogramming. As an ATP-dependent chromatin remodeling factor, the SWI/SNF complex collaborates with various lineage-determining factors (*e.g.,* PAX8, PPARγ) and stress response factors (*e.g.,* NRF2, HIF-1α) through the SWI/SNF complex's distinct subtypes (cBAF, PBAF, ncBAF). It occupies the promoter and enhancer regions of metabolism-related genes, thereby modulating chromatin accessibility and transcriptional activity. Studies have shown that SWI/SNF complex regulates key metabolic pathways such as glycolysis, the TCA cycle, OXPHOS, lipid metabolism, and oxidative stress through multiple mechanisms. Loss or functional disruption of SWI/SNF subunits induces metabolic reprogramming of specific pathways, exhibiting pathway dependence and cell specificity in metabolic adaptation. Therefore, the functional imbalance of SWI/SNF complex not only drives tumor metabolic plasticity but also provides potential intervention points for metabolic-targeted therapies.

Although most studies agree that SWI/SNF complex is involved in regulating key metabolic nodes, the SWI/SNF complex's role shows significant context-dependent effects, even exhibiting bidirectional roles. For example, as mentioned earlier, in TNBC, BRG1 collaborates with SRY-box transcription factor 4 to activate HK2, enhancing glycolysis (27). In contrast, in NSCLC, BRG1 promotes PKM2 dephosphorylation and maintains its tetramerization, inhibiting glycolysis and promoting OXPHOS (28). Similarly, in ccRCC, different subunit deletions lead to distinct metabolic effects: *PBRM1* deletion downregulates the ETC and promotes glycolysis (67), while *ARID1A* or *SMARCA4* deletion enhances OXPHOS dependency (44, 47). These differences may arise from the TF combinations recruiting SWI/SNF complex to different subsets of enhancers or from the complex subtypes (cBAF, PBAF, ncBAF) and subunit redundancy/replacement (*e.g.,* ARID1A↔ARID1B, SMARCA4↔SMARCA2), which rewrite the target gene profile and its metabolic effects.

When glycolysis is inhibited, tumor cells often rely on glutamine supplementation and OXPHOS to maintain energy supply. Therefore, theoretically, blocking GLS1 (*e.g.,* CB-839) or inhibiting complex I (*e.g.,* IACS-010759) could cut off the compensatory pathway. However, preclinical studies have shown that the efficacy varies significantly across different tumor types and genetic backgrounds: myeloid tumors are sensitive to GLS1 inhibition (77), while in melanoma and ccRCC, the efficacy is limited (78). This difference reflects the high plasticity of the metabolic network: when mitochondrial metabolism is impaired, cancer cells can reverse-activate glycolysis as an alternative pathway, thereby counteracting the effects of single-pathway inhibition. Therefore, targeting a single metabolic axis alone is unlikely to produce lasting efficacy; it is necessary to combine the blockade of compensatory mechanisms to achieve more stable synthetic lethality.

In addition to regulating tumor metabolism, the SWI/SNF complex is also closely associated with tumor cell growth, proliferation, and invasion, and can influence the tumor immune microenvironment through gene expression and metabolic pathway alteration. For instance, in gastric cancer and Oral Squamous Cell Carcinoma, high expression of BRG1 is significantly associated with vascular, lymph node, and distant metastasis as well as poorer survival prognosis (79, 80) melanoma, the loss of the PBAF complex enhances chromatin accessibility at IFN-γ response sites, upregulates the expression of chemokines such as CXCL9/CXCL10 and antigen presentation genes, thus promoting the infiltration and cytotoxic function of effector T cells, and enhancing tumor cell sensitivity to immune-mediated killing. These findings suggest that SWI/SNF complex defects may be associated with immune therapy responses (81).

Recent progress has advanced our understanding of the SWI/SNF complex's role in tumor metabolic reprogramming. However, many unresolved issues remain. This review highlights that different SWI/SNF subclasses may play distinct roles in various tumor types. Tumor heterogeneity further complicates this, as cancer, immune, and stromal cells within the same tumor can exhibit significant metabolic differences. Consequently, these cell populations may rely on different SWI/SNF subclasses to regulate specific metabolic pathways, leading to varied roles for the SWI/SNF complex in different tumor types and cell populations. Future research should integrate single-cell transcriptomics and chromatin accessibility sequencing to clarify the functions of the SWI/SNF complex in distinct tumor subgroups, immune cells, and stromal cells, revealing its microenvironmental dependence and spatial heterogeneity in metabolic networks.

Much of the current research, however, focuses on the roles of individual subunits or the overall SWI/SNF complex in regulating specific metabolic pathways, and how SWI/SNF dysfunction alters metabolic states in certain tumors. Notably, the three SWI/SNF subclasses (cBAF, PBAF, and ncBAF) show distinct chromatin localization preferences: cBAF predominantly binds to enhancer regions, while PBAF and ncBAF associate with promoters or specific chromatin regions. These differences likely influence the gene sets and metabolic pathways regulated by each subclass. Therefore, future studies should further explore the relationship between different SWI/SNF subclasses and specific metabolic pathways to determine whether these subclasses exhibit preferential regulation of particular metabolic routes. For instance, whether cBAF is more inclined to regulate glycolysis, PBAF to oxidative phosphorylation, *etc.* Moreover, the exchange of SWI/SNF subunits (*e.g.,* ARID1A ↔ ARID1B, SMARCA4 ↔ SMARCA2) between subclasses warrants further investigation to understand how this exchange affects their regulatory roles in metabolic pathways. This would provide insights into tumor-specific metabolic vulnerabilities and resistance mechanisms. Furthermore, constructing dynamic models that incorporate tumor evolution and drug intervention processes will be crucial in tracking the temporal changes between the SWI/SNF complex and metabolic pathways, identifying key nodes involved in metabolic adaptation and resistance formation. Exploring combined interventions targeting both epigenetic regulation and metabolic pathways could help mitigate the risk of tumor recurrence and metastasis, leading to more durable and precise therapeutic outcomes.

## Data availability

No data was used for the research described in the article.

## Conflict of interest

The authors declare that they have no conflicts of interest with the contents of this article.
